# Supplementary material for: Bactericidal Chitosan Derivatives and Their Superabsorbent Blends with ĸ-Carrageenan
Source: Int J Mol Sci. 2024 Apr 20;25(8):4534. doi: 10.3390/ijms25084534 (PMC11050674; doi:10.3390/ijms25084534)
Supplement: Supplementary file 1 [file ijms-25-04534-s001.zip › ijms-2955816-supplementary.pdf]

International Journal of *Molecular Sciences*

MDPI

*Supplementary Materials*

# Bactericidal Chitosan Derivatives and Their Superabsorbent Blends with κ-Carrageenan

**Kamila Lewicka [1], Anna Smola-Dmochowska [2], Natalia Śmigiel-Gac [2], Bożena Kaczmarczyk [2], Henryk Janeczek [2], Renata Barczyńska-Felusiak [1], Izabela Szymanek [1], Piotr Rychter [1] and Piotr Dobrzyński [1,2,*]**

1 Faculty of Science and Technology, Jan Dlugosz University in Czestochowa, 13/15 Armii Krajowej Av., 42-200 Czestochowa, Poland; k.lewicka@ujd.edu.pl (K.L.); r.barczynska-felusiak@ujd.edu.pl (R.B.-F.); izabela.szymanek@doktorant.ujd.edu.pl (I.S.); p.rychter@ujd.edu.pl (P.R.)
2 Centre of Polymer and Carbon Materials, Polish Academy of Sciences, 41-819 Zabrze, Poland; asmola@cmpw-pan.pl (A.S.-D.); ngac@cmpw-pan.pl (N.Ś.-G.); bkaczmarczyk@cmpw-pan.pl (B.K.); hjaneczek@cmpw-pan.pl (H.J.)
* Correspondence: p.dobrzynski@ujd.edu.pl

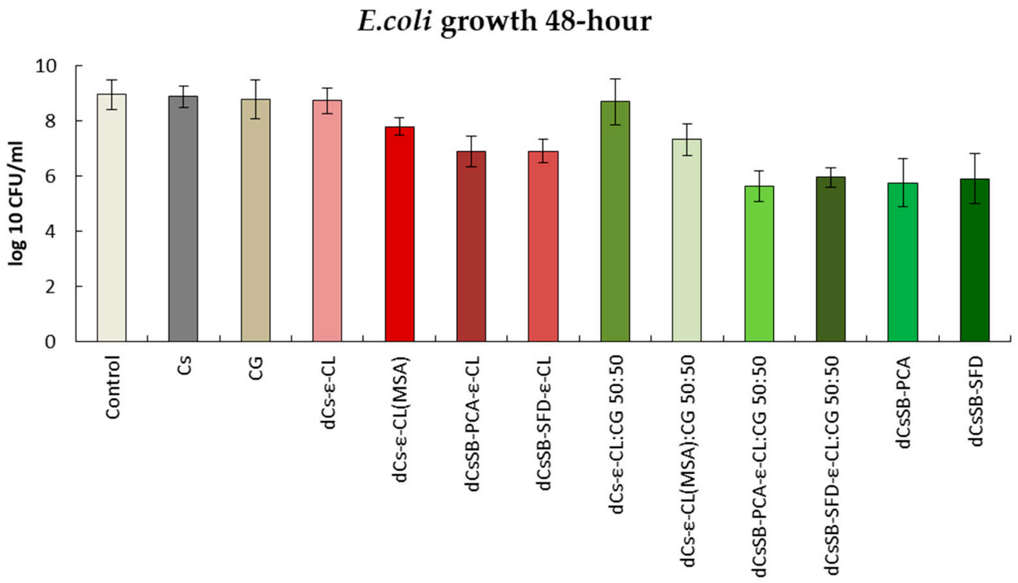


**Figure S1.** Summary of growth results for the *E. coli* for 48 h.

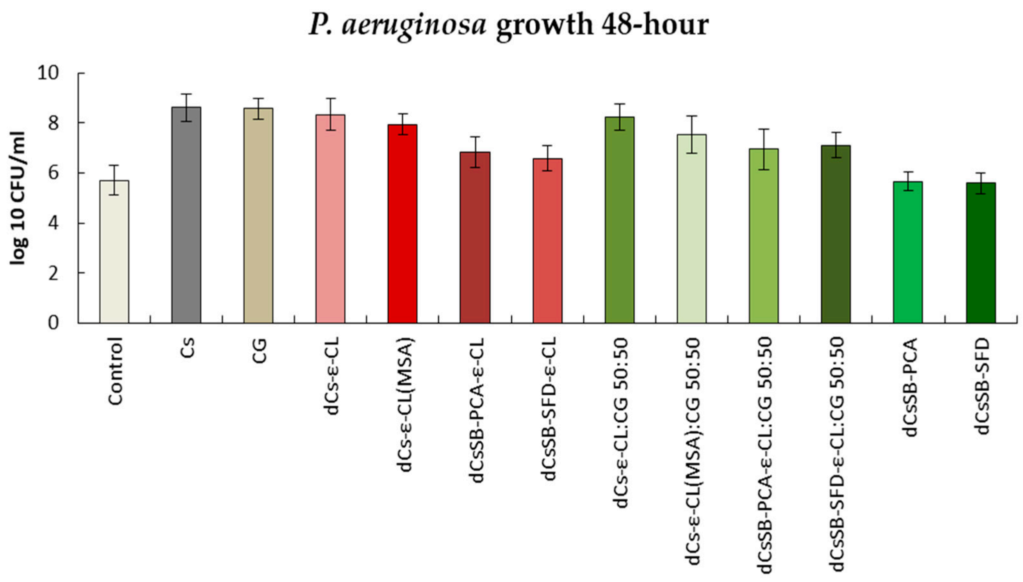


**Figure S2.** Summary of growth results for the *P.aeruginosa* for 48 h.

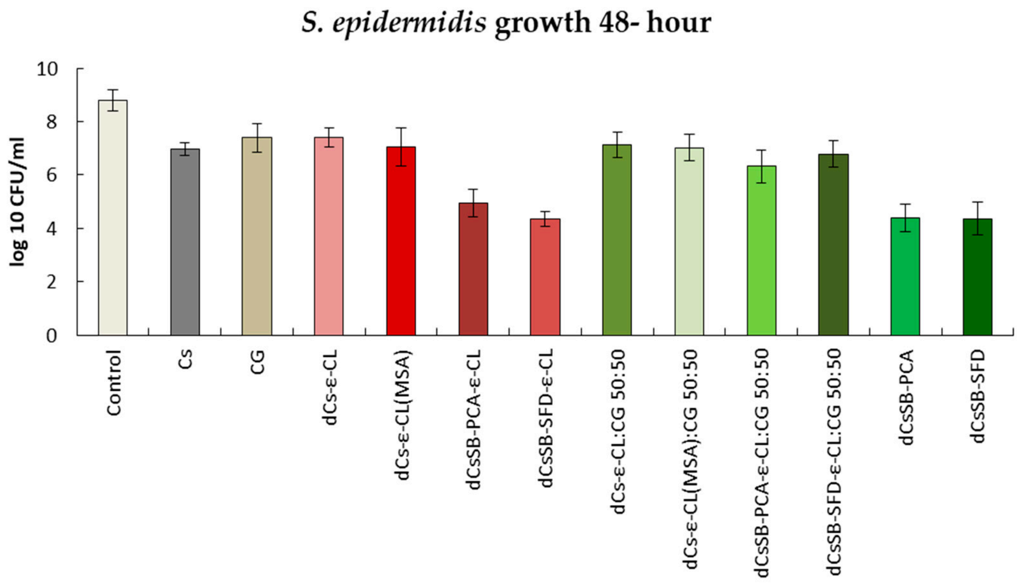


**Figure S3.** Summary of growth results for the *S. epidermidis* for 48 h.

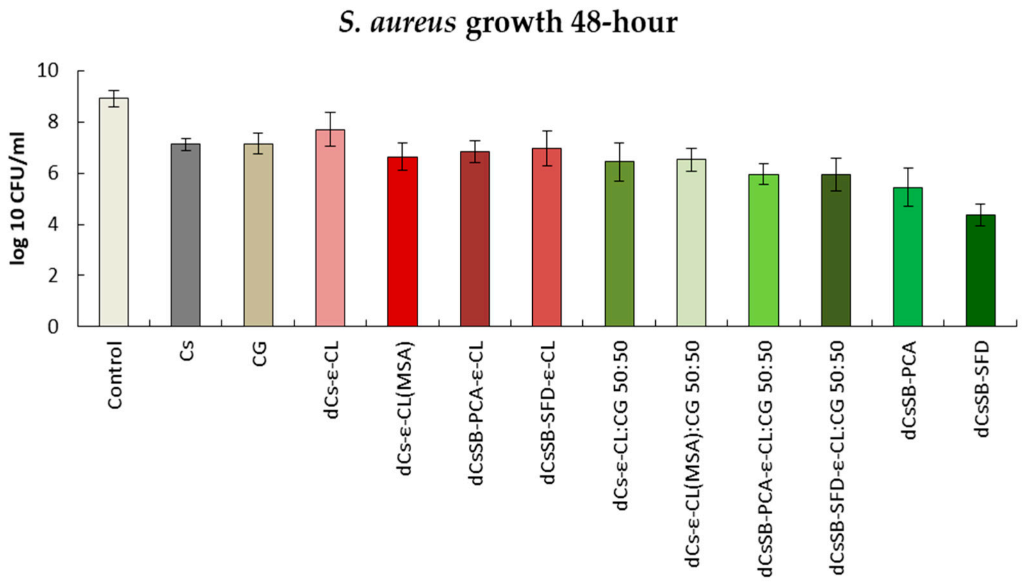


**Figure S4.** Summary of growth results for the *S. aureus* for 48 h.

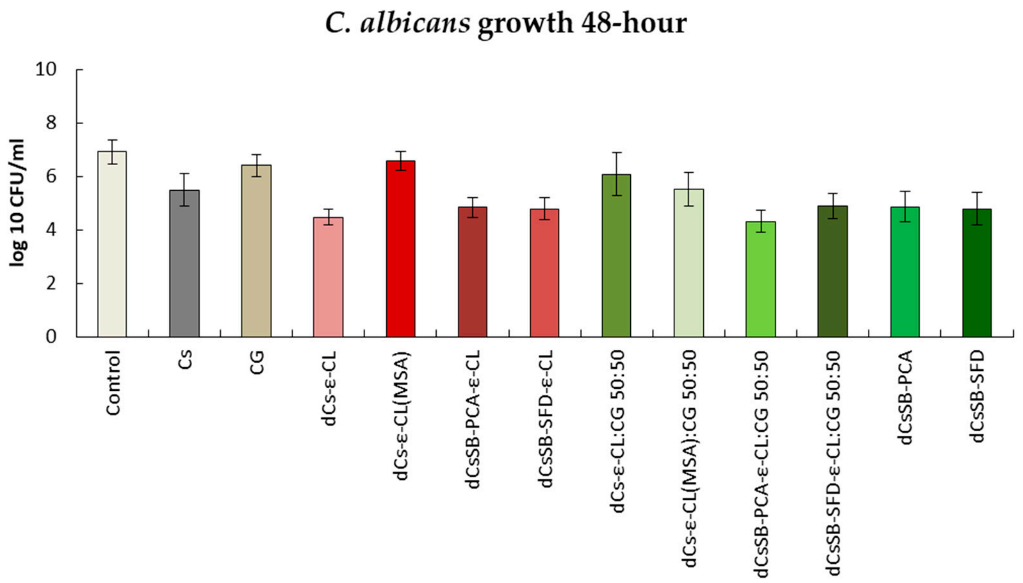


**Figure S5.** Summary of growth results for the *C. albicans* for 48 h.

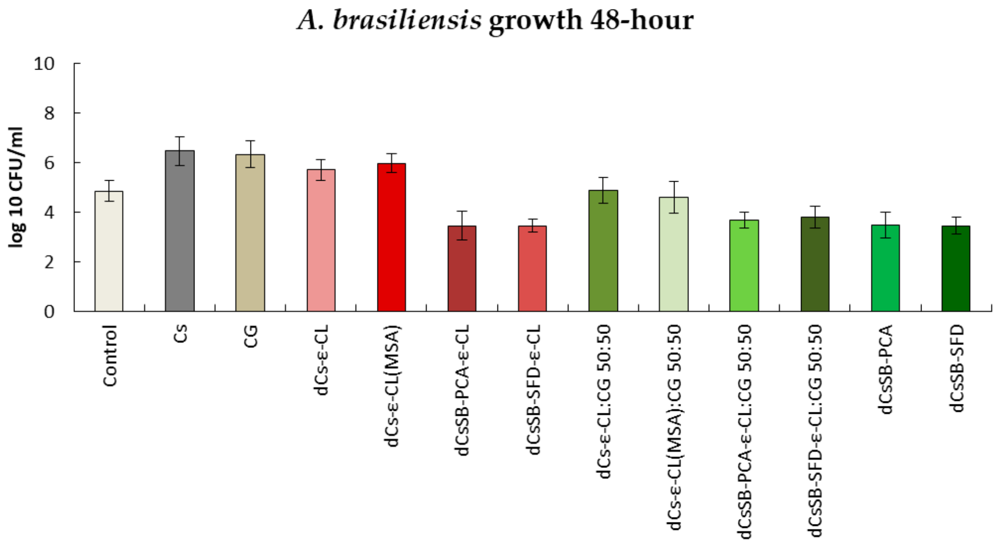


**Figure S6.** Summary of growth results for the *A. brasiliensis* for 48 h.
